# The prevalence of the fenestrated left renal vein

**DOI:** 10.1007/s00276-025-03614-y

**Published:** 2025-03-17

**Authors:** Nawwaf Sebastian Damen, Adelina Maria Jianu, Mugurel Constantin Rusu

**Affiliations:** 1 Department of Anatomy and Embryology, Faculty of Medicine, “Victor Babeș” University of Medicine and Pharmacy, Timișoara, 300041 Romania; 2 Division of Anatomy, Department 1, Faculty of Dentistry, “Carol Davila” University of Medicine and Pharmacy, Bucharest, 020021 Romania

**Keywords:** Kidney, Renal pedicle, Gonadal vein, Retropelvic tributary, Lumbar vein

## Abstract

**Purpose:**

The left renal vein (LRV) is typically a single preaortic vein. The discovery of fenestrated LRVs (FLRVs), a rare occurrence previously reported only twice, has piqued our interest. We aimed to determine the prevalence of such variants using an angioCT batch.

**Methods:**

We meticulously studied archived angioCT files of 95 men and 55 women. The morphology of the LRVs was carefully checked on planar sections and by three-dimensional volume renderings, ensuring the accuracy of our findings.

**Results:**

In 3.34% of cases, four males and one female were found FLRVs. Their posterior projections on the aorta were variable. The FLRVs were either partly preaortic, immediately to the left side of the aorta, or at a distance to the left side of the aorta. There were three true FLRVs, with a single vein attached at the lateral end and two pseudo-fenestrations, with two veins attached laterally. The left suprarenal and gonadal veins drained, respectively, in the superior and inferior arms of the fenestrations. In 2/5 cases, the second left lumbar vein drained into the inferior arms of the fenestrations. In one case, the FLRV was traversed by the inferior segmental branch of the renal artery.

**Conclusion:**

Our findings have significant implications for surgical procedures targeting the left renal pedicle. The FLRV, with its morphological and topographical variability, should be considered among the anatomical variations of the LRV that may impede or endanger such procedures. However, it can be accurately discriminated on angioCT scans, providing a potential solution to this challenge.

**Supplementary Information:**

The online version contains supplementary material available at 10.1007/s00276-025-03614-y.

## Introduction

Venous drainage of the kidneys is ensured by the left (LRV) and right (RRV) veins, typically single venous trunks. They end in the inferior cava vein. A retropelvic tributary (posterior primary tributary, posterior renal vein, supernumerary renal vein) of the LRV coursing posterior to the renal pelvis may exit the hilum and drain extrahillary into the LRV [9]. As the left renal pedicle is longer, the left kidney is preferred for donor nephrectomies [4]. Therefore, the variational anatomy of the LRV is subjected to numerous studies [2, 6].

Fenestrations are extremely rare in the venous system, especially in the renal veins [10]. A few cases were recently reported for the LRV fenestration (FLRV) [5, 10]. The fenestrations of the LRV are not listed in Bergman’s comprehensive Encyclopedia of Human Anatomic Variation [7].

We, therefore, aimed to study the prevalence of this rare morphology of the LRV.

## Material and method

Determinations were performed in a retrospective sample of 150 adult cases, 95 men and 55 women. Exclusion criteria were inadequate scans to observe the anatomy of the abdominal vessels, pathologic processes distorting the anatomical features, and previous retroperitoneal surgery. No cases were excluded. The research followed the principles of the World Medical Association Code of Ethics (Declaration of Helsinki). The Ethical Committee of the “Victor Babeş” University of Medicine and Pharmacy of Timişoara, Romania (affiliation 1) approved the study (approval no. 16178/11 July 2023).

The CT angiograms were performed with a 32-slice scanner (Siemens Multislice Perspective Scanner), with a 0.6 mm collimation and a reconstruction of 0.75 mm thickness with 50% overlap for a multiplanar maximum intensity projection and three-dimensional volume rendering technique, as described previously [3]. All the cases were documented using the Horos 3.3.6 (Horos Project, Annapolis, MD, USA) program. All the authors independently performed evaluations of the renal pedicles. The positive results were identical and were validated by each author. The cases with FLRVs were recorded and documented individually.

## Results

**Fig. 1:**
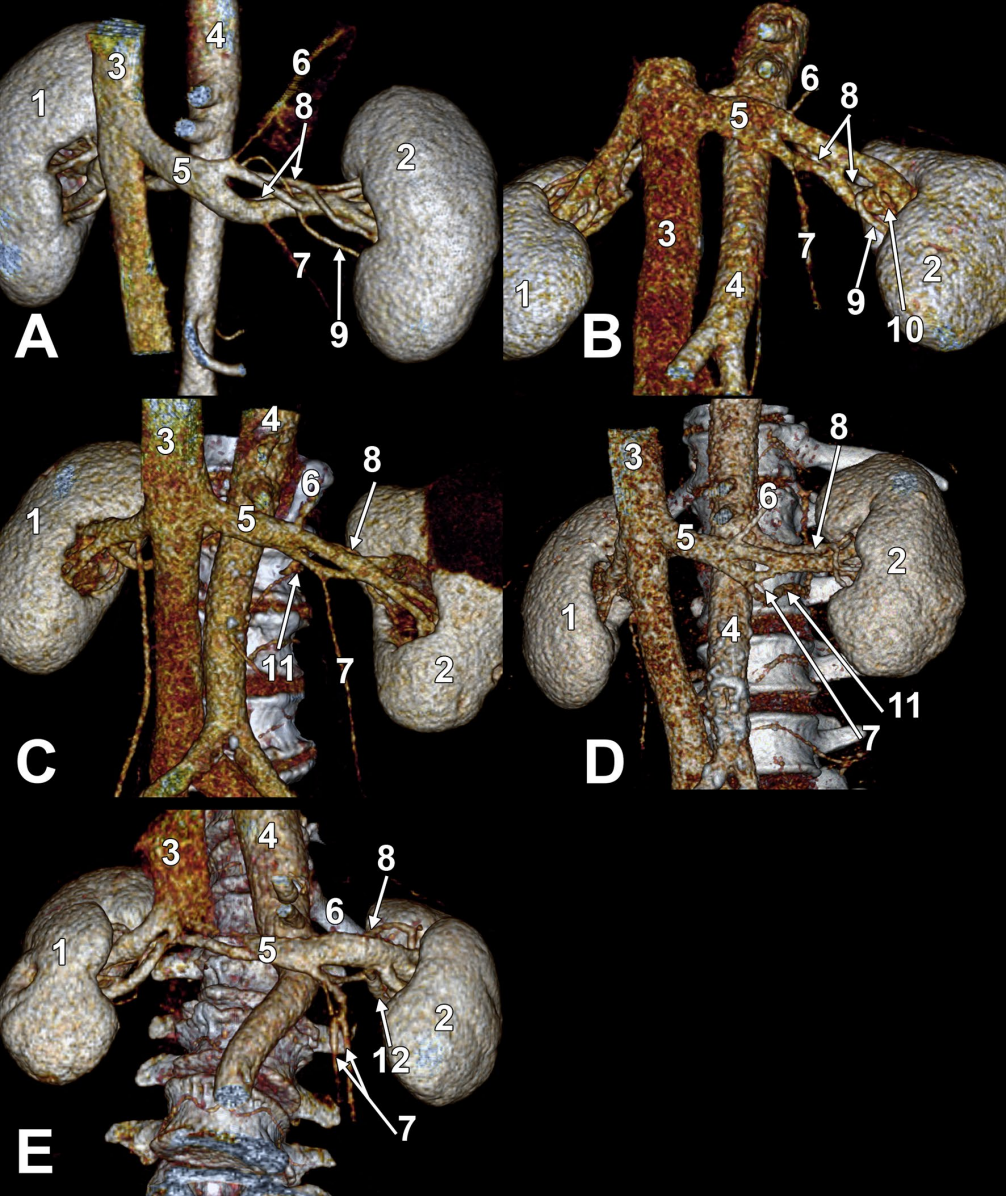
Five different cases (**A**–**E**) with fenestrations of the left renal vein. Three-dimensional volume renderings viewed anteriorly. 1. right kidney; 2., left kidney; 3. inferior cava vein; 4. abdominal aorta; 5. fenestrated left renal vein; 6. left suprarenal vein; 7. left gonadal vein (double in **E**); 8. left renal artery (double in **A**); 9. inferior segmental artery; 10. anterior inferior segmental artery; 11. second lumbar vein; 12. retropelvic tributary of the left renal vein

In 5/150 cases (3.34%), FLRVs were found (Online Resource 1). There were four male (Fig. 1A-D) and one female (Fig. 1E) cases. The calibre of the superior and, respectively, the inferior arm of the FLRV are listed in Table 1.

**Table 1:** The calibres of the superior and inferior arms of the left renal veins’ fenestrations

Case	Calibre (mm) of the superior arm of the FLRV	Calibre (mm) of the inferior arm of the FLRV	Figure
Male #1	4.78	11.6	1 A
Male #2	8.87	7.78	1B
Male #3	9.17	5.28	1 C
Male #4	8.85	4.64	1D
Female #5	13.01	7.26	1E

In cases #1, #2 and #5, the medial end of the LRV’s fenestration was in a sagittal plane tangent to the left side of the abdominal aorta. In case #3, the medial end of the LRV’s fenestration was far from the left side of the abdominal aorta. In case #4, the medial half of the LRV’s fenestration was applied on the anterior side of the abdominal aorta.

In all five cases of FLRVs, the superior arm of the fenestration received the left suprarenal vein and the inferior arm– the left gonadal vein (Fig. 1). In the female case #5, the left gonadal vein was double (Fig. 1E). In cases #3 (Fig. 1C) and #4 (Fig. 1D) the second left lumbar vein ended into the inferior arm of the LRV’s fenestration, medially to the upper end of the left gonadal vein.

In male case #1 (Fig. 1A), two left renal arteries emerged from the abdominal aorta. Each arm of the LRV’s fenestration had thus posteriorly one of the left renal arteries, superior and inferior. From the origin of the superior left renal artery, a long inferior segmental branch left. It traversed from postero-superior to antero-inferior the fenestration of the LRV.

In male case #2 (Fig. 1B), the LRV’s fenestration arms were later united by a short vertical trunk and continued towards the renal hilum as separate, superior and inferior LRVs. They were further twisted at the entrance into the renal hilum. In the female case #5 (Fig. 1E), the inferior arm of the LRV’s fenestration was joined by a retropelvic tributary before joining back the upper arm. In both cases, the lateral end of the fenestration attached two veins, not a single one. Therefore, this morphological pattern was regarded as a pseudo-fenestration. This is because it did not appear as a split through a single vessel.

## Discussion

Fenestration of the LRV is a rare anatomical variation that can have significant implications for surgical procedures and renal health. This condition involves a split or opening in the LRV, which can affect blood flow and complicate surgical interventions. It may result from the incomplete fusion of the embryonic circumaortic plexus.

**Fig. 2:**
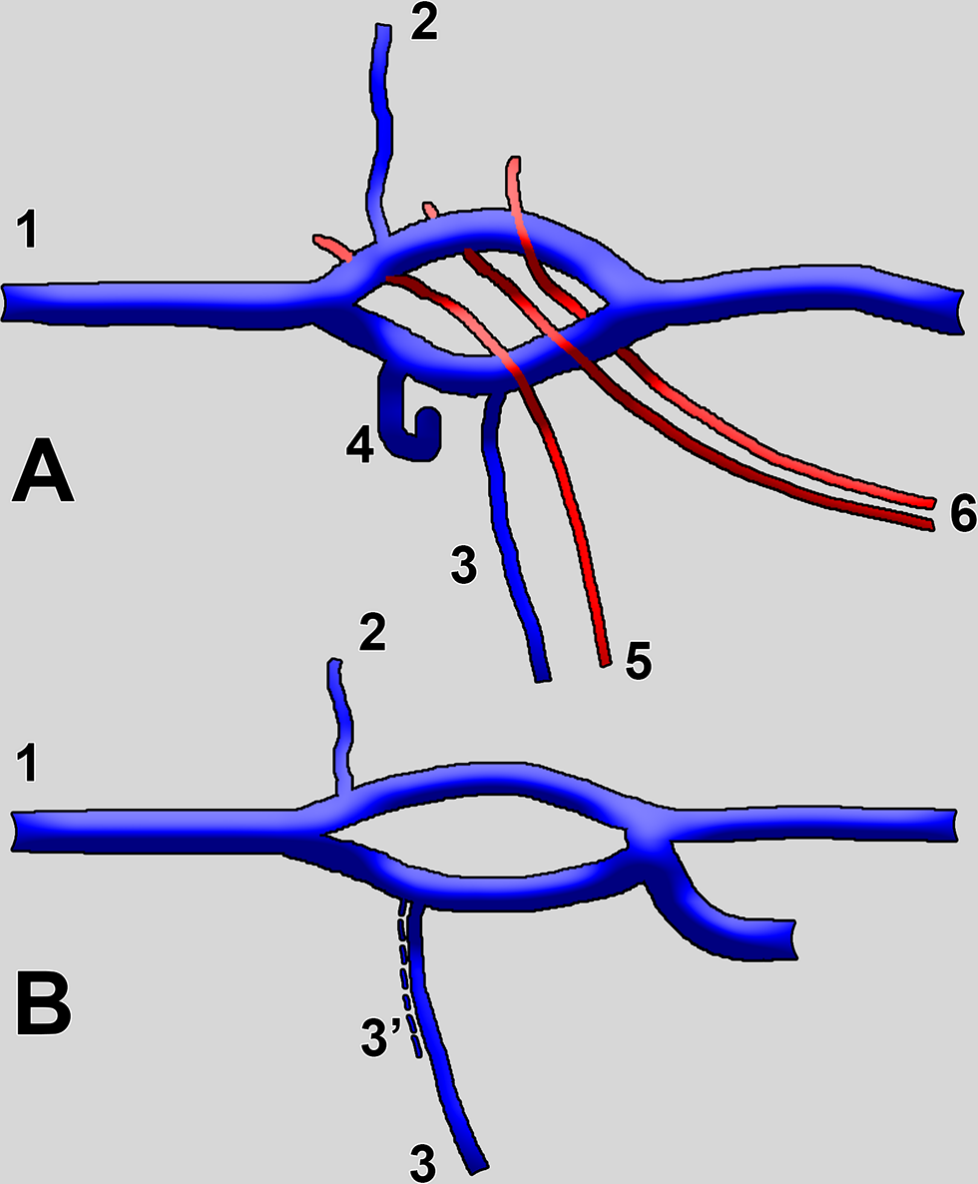
Diagram of the morphological patterns of the fenestrated (**A**) and pseudo-fenestrated (**B**) left renal vein. Fenestrations appear as a split of a single vessel. In pseudo-fenestrations, two distinctive veins join and contribute to the split. 1. left renal vein; 2. left suprarenal vein; 3. left gonadal vein, eventually duplicated (3’); 4. second left lumbar vein; 5. left gonadal artery; 6. inferior left segmental arteries

There were previously reported just two cases of FLRV [5, 10]. The calibres of the FLRV’s arms were not determined then. In the first one, found by angioCT in a male case, the left renal artery coursed above the FLRV and from it left the inferior segmental branch that traversed the LRV’s fenestration from anterior to posterior [5]. No branching pattern of the FLRV was detailed then. In one of our cases, a superior left renal artery coursed posterior to the fenestration’s upper arm and sent off an inferior segmental branch traversing from posterior to anterior the LRV’s fenestration. In the second previously reported case, the FLRV was found by dissection also in a male case [10]. The inferior arm of that fenestration received the left gonadal vein, and through that fenestration coursed from postero-superior to antero-inferior the left gonadal artery [10]. The second left lumbar vein draining into the inferior arm of a FLRV was not found previously. We found it in 2/5 FLRVs, constantly medial to the left gonadal vein, which is typical [1]. We also found the left suprarenal vein draining into the upper arm of LRV’s fenestration in 5/5 FLRVs. This detail was not observed or reported previously. In both previously reported cases, the LRV’s fenestration was at a distance on the left side of the aorta [5, 10]. We found here different topographical patterns: in 1/5 cases, the fenestration was partly applied on the anterior side of the aorta; in 3/5 cases, it was immediately on the left side of the aorta, and in 1/5 cases, it was at a distance to the left side of the aorta. Pseudo-fenestrations of the LRV were not found or reported previously.

A general diagram of the morphological possibilities results from the previous and present cases and is presented in Fig. 2.

A preaortic FLRV, as we found in 1/5 cases, may be subjected to a nutcracker phenomenon. In such a situation, fenestration may help discharge the venous blood pressure via the uncompressed arm of the fenestration and thus avoid congestion.

When performing venous sampling procedures of the left suprarenal vein (e.g. for hormonal assays), it should be selected the LRV that receives the adrenal vein, either the preaortic arm of a circumaortic LRV [8] or the superior arm of a FLRV.

Congenital variations of the LRV restrict its surgical availability for mobilisation procedures, such as the splenorenal shunts, and nullify the advantages which typically accrue from the greater length of the LRV (e.g. left renal transplant) [8]. Such is the case of an FLRV. In laparoscopic procedures with a limited field of vision and restricted possibilities for surgical manipulation [4], the morphology of the LRV should be carefully determined preoperatively because confusing an arm of a FLRV with a LRV may lead to severe bleeding and surgical discomfort. A FLRV we found here with a 3.34% prevalence should be distinguished from a double LRV that has been determined to have a 2.1% prevalence [6].

The incidence of the retropelvic tributary of the LRV ranges from 30.0 to 46.4%, which is very frequent [9]. However, a retropelvic tributary ending into a FLRV was not recorded previously in our knowledge.

Deák et al. (2011) found a lumbar vein draining into the LRV in 1/30 donor kidneys [4]. We found here 2/5 FLRVs receiving the second lumbar vein. The prevalence of the LRV drainage of the second lumbar vein may be higher [1].

## Conclusions

The FLRV should be considered among the anatomical variations of the LRV that may impede or endanger the surgical procedures targeting the left renal pedicle. Is can be accurately discriminated on angioCT scans.

## Electronic supplementary material

Below is the link to the electronic supplementary material.

Supplementary Material 1

## Data Availability

No datasets were generated or analysed during the current study.
